# Gut–Brain Crosstalk and the Central Mechanisms of Orofacial Pain

**DOI:** 10.3390/brainsci13101456

**Published:** 2023-10-13

**Authors:** Ran Tao, Sufang Liu, Joshua Crawford, Feng Tao

**Affiliations:** Department of Biomedical Sciences, Texas A&M University School of Dentistry, 3302 Gaston Ave., Dallas, TX 75246, USA

**Keywords:** gut microbiome, orofacial pain, vagus nerve, short-chain fatty acids, neurotransmitters, cytokines

## Abstract

Accumulated evidence has demonstrated that the gut microbiome can contribute to pain modulation through the microbiome–gut–brain axis. Various relevant microbiome metabolites in the gut are involved in the regulation of pain signaling in the central nervous system. In this review, we summarize recent advances in gut–brain interactions by which the microbiome metabolites modulate pain, with a focus on orofacial pain, and we further discuss the role of gut–brain crosstalk in the central mechanisms of orofacial pain whereby the gut microbiome modulates orofacial pain via the vagus nerve-mediated direct pathway and the gut metabolites/molecules-mediated indirect pathway. The direct and indirect pathways both contribute to the central regulation of orofacial pain through different brain structures (such as the nucleus tractus solitarius and the parabrachial nucleus) and signaling transmission across the blood-brain barrier, respectively. Understanding the gut microbiome-regulated pain mechanisms in the brain could help us to develop non-opioid novel therapies for orofacial pain.

## 1. Introduction

The gut microbiome, which includes a diverse array of microorganisms such as bacteria, archaea, fungi, and viruses that inhabit the digestive tracts of animals, has been found to play an important role in health and diseases [1,2,3]. Notably, the gut serves as the primary site for different microbiome compositions. The gut microbiome exerts a wide range of effects, including influencing colonization, enhancing resistance against pathogens, maintaining the integrity of the intestinal epithelium, metabolizing dietary and pharmaceutical compounds, regulating immune function, and even modulating behavior via gut–brain crosstalk. Bidirectional communication between the gastrointestinal tract and the brain, particularly involving the gut microbiome, has increasingly been recognized as the gut microbiome–gut–brain axis. In recent years, it has been proposed that the gut microbiome–gut–brain axis exists across the lifespan [4]. Gut microbiome metabolites can affect brain functions through the vagus nerve and different signaling pathways [5]. This intricate relationship includes multiple afferent and efferent components that play important roles in regulating homeostatic processes such as satiety, hunger, inflammation, and pain. Accumulating evidence has demonstrated associations between the gut microbiome and neurological disorders, including different types of pain [6,7,8,9]. Orofacial pain is highly prevalent with debilitating pain conditions in the oral cavity, head, and face, and its pathogenesis is still not fully understood. Orofacial pain has profound implications for various important aspects of individuals’ lives, including aesthetics, speech, eating, and psychosocial well-being. Orofacial pain affects approximately 25% of the population [10], and management of such pain remains to be a challenge for clinicians and dentists. In this review, we discuss the relevant gut microbiome metabolites/molecules involved in orofacial pain, and we summarize recent studies that have investigated potential mechanisms underlying the roles of the gut microbiome and gut–brain crosstalk in orofacial pain.

## 2. Role of the Gut Microbiome in Orofacial Pain

Migraine is a chronic neurological disorder that has a negative impact on the quality of life among migraineurs. A metagenome-wide association study has shown significantly lower alpha diversity at the species, genus, and Kyoto Encyclopedia of Genes and Genomes orthologous levels, in elderly women with migraine compared with a matched healthy control group [11]. The migraine group was enriched in kynurenine degradation and γ-aminobutyric acid (GABA) synthesis; however, the healthy controls had higher glycolysis, homoacetogenesis, quinolinic acid degradation, and S-adenosyl methionine synthesis [11]. This study identified differences in gut microbiome composition and functional profiling between the migraine and healthy groups, which could provide novel therapeutic targets for developing effective migraine treatment. Another clinical study showed associations of the gut microbiome with migraines in children [12]; higher abundances in the genus of phyla *Bacteroidetes*, *Actinobacteria*, *Firmicutes*, and *Proteobacteria* were observed in children with migraine than those in children without migraine [12]. In animal experiments, 16S rRNA sequencing has revealed that the abundance of *Firmicutes* was significantly lower, whereas the abundance of *Bacteroides* was much higher, in rats with nitroglycerin (NTG)-induced chronic migraine compared with a control group [13]. In our previous study [14], we found that gut colonization with fecal microbiome transplantation (FMT) completely prevented the prolongation of NTG-induced migraine-like pain in germ-free mice and that tumor necrosis factor alpha (TNFα) in the spinal trigeminal nucleus caudalis (Sp5C) may contribute to the gut microbiome-produced regulation of migraine-like pain. Furthermore, a recent study using germ-free mice [15] suggested the involvement of the gut microbiome in migraine modulation by showing that germ-free mice receiving gut microbiome from a migraine patient developed more severe NTG-induced hyperalgesia as compared with germ-free mice receiving gut microbiome from a healthy control [15].

Temporomandibular disorder (TMD) is characterized with facial pain in the temporomandibular joint (TMJ), masticatory musculature, and related structures, and it affects 5–12% of the population [16,17]. It has been reported that the gut microbiome composition is associated with joint pain [18]. The gut microbiome can regulate both innate and adaptive immune systems, which become dysregulated in gut dysbiosis, and then trigger joint pain. In our recent study [19], we revealed that the levels of gut bacteria *Bacteroidetes* and *Lachnospiraceae* were significantly reduced in a complete Freund’s adjuvant (CFA)-induced TMJ pain mouse model, and we also found that resveratrol, a natural bioactive compound with anti-inflammatory properties, dose-dependently inhibited such TMJ pain and reversed CFA-caused reduction of the two gut bacteria. Moreover, FMT with feces from resveratrol-treated mice has been shown to significantly diminish CFA-produced TMJ inflammation, blood-brain barrier (BBB) leakage, microglial activation, and TNFα release in the Sp5C [19].

A tension-type headache is a common primary headache that often causes mild-to-moderate pain around the head, face, or neck [20]. Similar to migraine, tension-type headaches are more prevalent in women. A clinical study showed a higher level of constipation in women with tension-type headaches [21]. A link between constipation and headache has also been observed in children and adolescents [22,23]. These clinical investigations suggest that there is a causal relationship or a common pathogenesis between the two clinical conditions. It has been shown that the gut microbiome plays an important role in the regulation of constipation [24,25]. Lin et al. observed a significant alteration in the abundance and diversity of the gut microbiome in constipated mice, and specifically, they showed an increase in *Prevotella*, *Ruminococcus*, and *Turicibacter* and a decrease in *Lactobacillus* in the mice with constipation [26]. Taken together, these studies indicate that one of the potential mechanisms for tension-type headache could be related to altered gut microbiome.

Dental pain is caused by lesions or diseases that affect the teeth and/or their immediate and supporting structures, mainly including endodontitis and periodontitis [27]. Although there is no direct evidence to indicate the role of the gut microbiome in dental pain, there is abundant indirect information that suggests the gut microbiome affects endodontitis and periodontitis via the gut–bone axis or the oral–intestinal–brain axis, which may have an impact on secondary pain in these disorders [28,29].

## 3. The Role of Gut Microbiome Metabolites and Gut-Releasing Molecules in Orofacial Pain

### 3.1. Short-Chain Fatty Acids (SCFAs)

SCFAs are mainly produced by the gut microbiome through bacterial fermentation of nondigestible carbohydrates and they include acetic acid, propionic acid, and butyric acid [30]. SCFAs can enter the peripheral blood circulation, and then cross the BBB into the central nervous system (CNS). SCFAs modulate neuropathic pain by regulating microglial activation and subsequent proinflammatory phenotypic polarization [31]. It has been reported that SCFAs inhibited microglial activation and reduced the release of inflammatory factors via the microbiome–gut–brain axis [32]. Specifically, SCFA butyrate can act as a histone deacetylase inhibitor to regulate intestinal macrophage function, modulate periodontal mechanical nociception via SCFA receptor signaling, and attenuate neuropathic pain by its anti-inflammatory action [33,34,35]. In an NTG-induced migraine mouse model, SCFA treatment with sodium propionate and sodium butyrate attenuated migraine-like pain, reduced histological damage in the trigeminal nucleus, decreased the expression of proinflammatory mediators, and alleviated related intestinal alterations [36]. Further investigation with 16S rRNA sequencing has indicated that such SCFA treatment could restore the intestinal microbiome profile and exert a protective effect on perturbation of the gut microbiome in the migraine condition [37]. Knox et al. reported that the two SCFAs butyrate and propionate increased tight junction protein spikes, improved BBB integrity, and modulated mitochondrial network dynamics [38], which suggests that SCFAs play a critical role in actin cytoskeletal rearrangement and BBB function. In addition, SCFAs have been shown to alleviate the loss of oligodendrocyte precursor cells by inhibiting astrocyte activation [39].

Our recent study [19] using a CFA-induced TMJ pain mouse model revealed that intra-TMJ CFA injection not only induced persistent joint pain, but also reduced SCFA levels and decreased SCFA-producing bacteria in the gut, and resveratrol reversed the CFA-caused reduction of SCFAs and SCFA-producing gut bacteria. These results suggest that SCFAs and those SCFA-producing bacteria could be targeted to develop a novel pain therapy. However, which receptor mediates the effect of SCFAs on orofacial pain remains unclear. We plan to investigate the receptor mechanism and related downstream pathways in the future. Further studies will help us to better understand the underlying mechanisms for orofacial pain.

### 3.2. Neurotransmitters

The gut microbiome can produce a variety of neurotransmitters, which mainly include serotonin (5-HT), dopamine, norepinephrine, and GABA. These neurotransmitters can influence neural signaling through the microbiome–gut–brain axis. It has been reported that the turnover rates of three monoamines (norepinephrine, dopamine, and 5-HT) in the striatum are much higher than those in specific pathogen-free control mice [40]. More and more evidence has demonstrated that these neurotransmitters contribute to the modulation of orofacial pain.

Approximately 95% of 5-HT is produced in the gut [41]. 5-HT signaling is critical for descending modulation of orofacial pain [42,43]. Okubo et al. reported that the maintenance of secondary hyperalgesia in a chronic constriction injury of the trigeminal infraorbital nerve (CCI-ION)-induced neuropathic pain model depended on the descending 5-HT pathway from the rostral ventromedial medulla and activation of 5-HT3 receptors in the Sp5C [44]. Tryptophan is the precursor of 5-HT, and approximately 90% of tryptophan is metabolized along the kynurenine pathway, through which tryptophan in the gastrointestinal tract is converted to kynurenine. The rate of tryptophan metabolism depends on the expression of indoleamine-2,3-dioxygenase and tryptophan-2,3-dioxygenase [45,46]. In addition, secretion of small molecules (such as SCFAs and bile acids) in the gut can signal enterochromaffin cells to produce 5-HT through the expression of tryptophan hydroxylase [47]. In addition, Mustafa et al. observed that 5-HT concentrations were significantly increased in the spinal trigeminal nucleus oralis and interpolaris as well as the nucleus tractus solitarius (NTS) in a mild closed head traumatic brain injury rat model, suggesting that 5-HT signaling in the trigeminal system is involved in the modulation of orofacial sensation [48].

The L-dopa produced by gut bacteria can enter the CNS through the blood circulation, and then is transformed to dopamine. Wang et al. observed that berberine increased the production of L-dopa in the gut and dopamine in the brain to ameliorate Parkinson’s disease by regulating the gut microbiome [49]. A clinical study using FMT suggested that dopamine and 5-HT transporters in the brain could be regulated by the gut microbiome [50]. Meanwhile, many studies have demonstrated that dopamine signaling contributed to the modulation of orofacial pain. Shamsizadeh et al. reported that dopamine receptors in the dorsal hippocampus were involved in the inhibition of formalin-induced orofacial pain [51]. The plasma dopamine level has been suggested to serve as a biomarker for myofascial TMD pain [52]. Nigrostriatal dopaminergic pathway depletion has been shown to produce orofacial static mechanical allodynia in a CCI-ION neuropathic pain rat model [53] and to induce trigeminal dynamic mechanical allodynia in a Parkinson’s disease rat model [54]. Another study [55] also showed that depletion of the nigrostriatal dopaminergic pathway may be associated with noxious stimulation-produced hypersensitivity in the orofacial region. The dopamine receptors in the ventral tegmental area have been shown to be involved in antinociceptive effects produced by lateral hypothalamus stimulation in an orofacial pain rat model [56]. In our study, we revealed that the dopamine receptors D1 and D2 in the anterior cingulate cortex (ACC) had opposite roles in the modulation of trigeminal neuropathic pain: optogenetic activation of D1-expressing neurons in the ACC significantly exacerbates CCI-ION-induced trigeminal neuropathic pain in both early and late phases, whereas optogenetic activation of D2-expressing neurons in the ACC strongly ameliorated such pain in the late phase [57]. We previously showed that specific excitation of dopaminergic neurons in the hypothalamic A11 nucleus attenuated trigeminal neuropathic pain by activating the D2 receptors-mediated descending pain modulation pathway [58].

Norepinephrine has been found to be an essential regulator of the gut–brain axis. In addition, it contributes to the regulation of the following functions in the gastrointestinal tract: blood flow, gut motility, nutrient absorption, and interaction with innate immune system [59]. An in vitro study observed that *E. coli*, *Proteus vulgaris*, *Serratia marcescens*, *Bacillus subtilis*, and *Bacillus mycoides* could harbor relatively high levels of norepinephrine in their biomass [60]. It has been reported that norepinephrine could be produced as a quorum-sensing molecule in bacteria [61]. In germ-free mice, the level of norepinephrine in the cecal lumen was significantly decreased, which could be recovered by gut microbiome colonization or transplantation with a mixture of 46 *Clostridia* species [62]. These results strongly indicate that the gut microbiome influences the level of norepinephrine in the cecal lumen; however, whether gut bacteria directly produce norepinephrine or indirectly increase the level of norepinephrine in the gut by modulating host production remains to be investigated. Administration of reboxetine, a selective norepinephrine reuptake inhibitor, has been show to perturb the gut microbiome, including a low *Firmicutes*/*Bacteroidetes* ratio and low *Lactobacillus* level in rats [63]. Norepinephrine-mediated signal transmission in the ventral part of the bed nucleus of the stria terminalis is involved in bidirectional gut–brain interactions [64]. It has been reported that GABA_A_-mediated activation of norepinephrine neurons in the medial prefrontal cortex enhanced hypersensitivity via α1 receptors, in a CCI-ION-induced trigeminal neuropathic pain rat model [65].

In addition, GABA and GABA receptors are involved in gastrointestinal tract motility [66]. It is known that a broad diversity of bacteria is able to produce GABA. Interestingly, the production of GABA by bacteria has a physiological purpose, in other words, secretion of GABA serves as a mechanism to decrease intracellular pH via the glutamate acid resistance system [67]. The gut microbiome is able to influence circulating GABA, because the levels of GABA in the lumen and serum of germ-free animals are dramatically reduced [68]. Several commensal bacteria have been reported to produce GABA, including members of the *Bifidobacterium* and *Lactobacillus* genera. A previous study found that oral supplementation of *Bifidobacterium breve* NCIMB8807 pESHgadB, which has been engineered to produce GABA via overexpression of glutamate decarboxylase B, could diminish sensitivity to visceral pain in a rat model [69]. More importantly, supplementation with *Bifidobacterium breve* NCIMB8807, the wild-type strain of the bacteria, had no effect on visceral pain behavior, suggesting that the analgesic effect is due to the engineered bacteria-produced GABA [69]. GABA signaling in the ganglia and CNS contributes to pain modulation. Specifically, GABA_B_ receptor activation attenuates inflammatory orofacial pain by modulating interleukin-1β in satellite glial cells [70]. The GABA_A_ receptor-mediated inhibitory system in the central nucleus of the amygdala can also modulate pain and itch behaviors, and a selective GABA_A_ receptor agonist can inhibit both pain and itch responses [71].

### 3.3. Cytokines

Cytokines are small molecular peptides or glycoproteins that are synthesized and secreted by various tissue cells, mainly immune cells [72]. Cytokines mediate cellular interactions to release a variety of inflammation-regulating proteins when pathogens are present in the body, which can stimulate and recruit immune cells [73,74]. Many studies have shown that dysbiosis of the gut microbiome in patients with different diseases leads to abnormal secretion of cytokines [75,76,77,78]. Gut microbiome and serum metabolome analyses have identified a reduction in gut bacterial diversity and cytokines as potential molecular biomarkers in fibromyalgia [79]. In a retrospective cohort study, Wang et al. reported that long-term oral administration of oxycodone induced abnormal changes in cytokine levels and intestinal flora in patients with moderate to severe cancer pain and that analgesic tolerance caused by long-term use of oxycodone may be closely associated with sustained upregulation of interleukin-6 and TNFα levels, which can simultaneously contribute to a chronic systemic inflammatory response [80].

In our studies, we have demonstrated that gut microbiome dysbiosis contributes to the chronicity of migraine-like pain by upregulating TNFα in the trigeminal nociceptive system [14], and we have also revealed that treatment with resveratrol restores the BBB integrity, inhibits microglial activation, and decreases the release of TNFα in the Sp5C [19]. In addition, a previous study showed that TNFα dysregulation promoted cytokine proteome profile changes and microglial activation in bilateral spinal trigeminal nucleus, which contributed to bilateral mechanical hypersensitization in a chronic trigeminal neuropathic pain mouse model [81].

## 4. Role of Gut–Brain Crosstalk in the Central Regulation of Orofacial Pain

### 4.1. Vagus Nerve-Mediated Direct Pathway for Gut–Brain Interactions

The vagus nerve can transmit signals from the gut to the brain, which is an important communication channel for gut–brain crosstalk [82,83,84]. The sensory information originating from the gut is conveyed via vagus nerve sensory fibers that terminate in the nodose ganglia, where the incoming sensory signals are processed. Subsequently, the processed signals are transmitted to the CNS, specifically to the NTS in the medulla oblongata of the brainstem [85]. The NTS serves as a key relay center for sensory information from various visceral organs, including the gut, and plays crucial roles in regulating autonomic and homeostatic functions in the body [86]. Invasive cervical vagus nerve stimulation has been developed rapidly for the treatment of orofacial pain, especially migraine [87,88,89]. Okada et al. reported that enhanced noxious inputs from the NTS to the parabrachial nucleus (PBN) modulated PBN neuron activity and contributed to the affective component of trigeminal neuropathic pain in a CCI-ION rat model [90], which suggests that the NTS-PBN pathway may be a potential therapeutic target for such orofacial pain. In addition, the NTS projects to the central nucleus of the amygdala (CeA) and the NTS-CeA pathway has been shown to modulate depression-like behaviors in chronic pain [91]. Moreover, the orofacial pain primary center Sp5C can project to the NTS; however, it remains unknown how the neural circuit Sp5C-NTS-PBN/CeA is involved in the vagus nerve-mediated central regulation of orofacial pain.

Additionally, gastric signals transmitted by the vagus nerve may alter the function of the amygdala, and then regulate emotion and pain behaviors [92]. Krieger et al. observed that vagal sensory signals from the gastrointestinal tract were critical for feeding-induced tuning of anxiety via the CeA in rats [93]. The CeA can modulate gut-related neurons in the dorsal vagal complex, which indicates a neural circuit mechanism for the regulation of gastrointestinal activity by the CeA [94]. Further studies are needed to demonstrate the role of amygdala, especially CeA, in vagus nerve-linked gut–brain crosstalk and its regulation of orofacial pain.

### 4.2. Indirect Pathways for Gut–Brain Interactions

In addition to vagal nerve-mediated direct gut–brain interactions, indirect pathways mediated by the gut microbiome metabolites and gut-releasing molecules, previously discussed, also contribute to gut–brain interactions through signaling transmission across the BBB. Specifically, bacterial lipopolysaccharides (LPS) from the gut can be delivered to the brain, can stimulate neuroinflammation, and then cause nerve injury in the brain [95], which is consistent with other studies regarding the effect of gut LPS on BBB integrity and brain function [96,97,98]. LPS play an essential physiological role in the gut, where they regulate the immune system and maintain gut barrier function [99,100]. Specifically, LPS are a main constituent of the Gram-negative bacterial membrane and activate Toll-like receptor 4, thereby leading to the production of pleiotropic cytokines/chemokines. LPS released by gut microbiome would be able to make a great impact on gut homeostasis via the intracellular signaling pathways engaged by host–microbiome interactions. In an orofacial pain mouse model with lip injection of LPS, the central microglia were activated and contributed to inflammation and pain development [101]. LPS mainly bind to Toll-like receptor 4, and this receptor blockade in the trigeminal ganglion can attenuate facial thermal and mechanical nociceptive sensitization induced by LPS [102]. In a CCI-ION rat model, local injection of LPS enhanced CCI-ION-induced trigeminal neuropathic pain, increased interleukin-1β in the ipsilateral trigeminal ganglion, and upregulated inducible nitric oxide synthase in the ipsilateral Sp5C [103], suggesting that LPS-mediated inflammatory priming is involved in the development and maintenance of such pain through interleukin-1β and inducible nitric oxide synthase signaling in the trigeminal nociceptive system.

## 5. Conclusions

**Figure 1:**
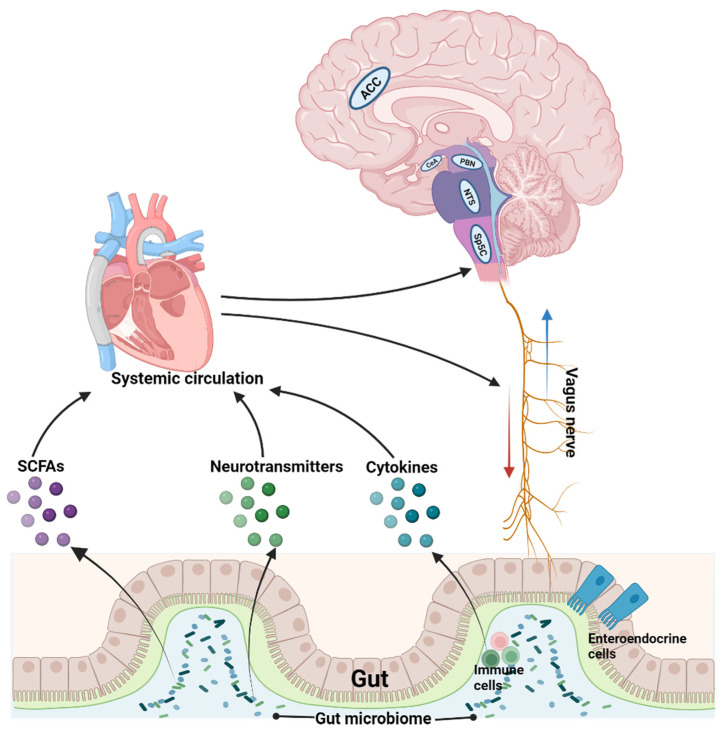
Gut microbiome contributes to the central regulation of orofacial pain through vagus nerve-mediated direct gut–brain crosstalk and gut metabolites/molecules-mediated indirect gut–brain crosstalk via systemic circulation. ACC, anterior cingulate cortex; CeA, central amygdala; NTS, nucleus tractus solitarius; PBN, parabrachial nuclei; Sp5C, spinal trigeminal nucleus caudalis.

In summary, the gut microbiome contributes to modulation of orofacial pain through vagus nerve-mediated direct gut–brain crosstalk and gut metabolites/molecules-mediated indirect gut–brain crosstalk via systemic circulation (Figure 1). The discovered information provides new insights into the understanding of orofacial pain and can help to develop novel potential therapies for such pain by targeting relevant gut microbiome metabolites. Although more and more evidence has indicated the involvement of the gut microbiome in different types of pain including orofacial pain, further studies are needed to provide detailed information regarding the specific roles of different species of gut bacteria in pain modulation and how the identified gut microbiome-related mechanisms are integrated with the existing pain signaling pathways in the CNS. In addition, systemic disease-caused changes in oral microbiota may produce intestinal dysbiosis and neuroinflammation [104], and oral pathobionts may promote neuroinflammation in the brain [105], which could also be involved in the pathogenesis of orofacial pain. Taken together, these exciting clues not only provide possible directions for future research to unravel the relationship between orofacial pain and gut–brain crosstalk, but also offer potential opportunities to develop personalized therapies for orofacial pain based on individuals’ gut microbiome profiling and relevant metabolites in the gut.

## Data Availability

Not applicable.
